# Comparison of intradialytic neuromuscular electrical stimulation and oral nutritional supplements in hemodialysis patients: study protocol for a multicenter, parallel-group, randomized controlled trial in Korea

**DOI:** 10.1186/s13063-021-05918-x

**Published:** 2021-12-20

**Authors:** Mi-yeon Yu, Jae Hyeon Park, Yong Chul Kim, Jae Yoon Park, Ran-hui Cha

**Affiliations:** 1 Department of Internal Medicine, Hanyang University Guri Hospital, Guri, Gyeonggi-do, Republic of Korea; 2 Department of Rehabilitation Medicine, Hanyang University Guri Hospital, Guri, Gyeonggi-do, Republic of Korea; 3 Department of Internal Medicine, Seoul National University Hospital, Seoul, Republic of Korea; 4 Department of Internal Medicine, Dongguk University Ilsan Hospital, Ilsan, Gyeonggi-do, Republic of Korea; 5 Department of Internal Medicine, National Medical Center, 245, Eulji-ro, Jung-gu, Seoul, 04564 Republic of Korea

**Keywords:** Hemodialysis, Neuromuscular electrical stimulation, Muscle strength, Physical function, Protein supplementation, Quality of life

## Abstract

**Background:**

The prevalence of sarcopenia increases as renal function decreases, and a considerable number of hemodialysis (HD) patients have sarcopenia. Exercise and nutritional support are established interventions to prevent and treat sarcopenia. Recently, many studies evaluating intradialytic neuromuscular electrical stimulation (NMES) showed improvement of muscular strength and mass, functional capacity, and quality of life (QOL). However, there has been no research about the effect of simultaneous nutritional support and NMES in HD patients.

**Methods:**

This is a 12-week, randomized controlled, parallel-group, multicenter trial of intradialytic NMES and protein supplementation for HD patients. Seventy-two patients receiving HD will be randomly assigned in a 1:1:1:1 ratio to control, intradialytic NMES only, protein supplementation only, and intradialytic NMES combined with protein supplementation groups. NMES will be delivered to a total of four areas of the bilateral vastus medialis and vastus lateralis using a 4-channel NMES instrument. A total of 25 g of protein supplements will be provided at the beginning of every dialysis session or after the NMES. The primary endpoint is the difference of hand grip and leg muscle strength at 12 weeks among 4 treatment groups. Secondary endpoints include muscle mass, physical performances, and questionnaires about QOL and physical activity.

**Discussion:**

In this study, we will evaluate the differential effectiveness of nutritional support and NMES during HD on muscle strength, muscle mass, physical function, and QOL. We expect that this study can provide guidelines for a new therapeutic option for HD patients who are unable or hesitant to exercise. Furthermore, this option can offer an opportunity to improve the physical function, QOL, and prognosis of HD patients.

**Trial registration:**

Clinical Research Information Service (CRIS), Korea, KCT0005573. Retrospectively registered on 03 November 2020

## Introduction

Hemodialysis (HD) is a type of renal replacement therapy that removes fluid, electrolytes, and uremic toxins that have accumulated in the body due to reduced kidney function. There are approximately 80 thousand (1497.6 patients/million population) end-stage renal disease (ESRD) patients receiving HD in Korea, and the number of patients has been increasing by 5–8% annually.

Sarcopenia is characterized by loss of muscle mass and strength, and decreased quality of life (QOL) with age, morbidities, and immobility and is associated with protein-energy wasting (PEW) in chronic kidney disease (CKD) and ESRD patients [1]. Sarcopenia is also associated with functional decline, increased risk of falling, and increased mortality in CKD and ESRD patients [2, 3]. In addition, sarcopenia in dialysis patients is associated with depression leading to decreased survival [4].

The prevalence of sarcopenia in HD patients varies from 21 to 68% depending on the methodology and demographics [5]. CKD-associated catabolic alterations can explain the profound features of sarcopenia in CKD patients [6]. CKD is associated with chronic low-grade inflammation leading to progressive weight loss, muscle weakness, and loss of the ability to exercise. Inflammatory cytokines and inactivity-mediated destruction of protein homeostasis result in the catabolic destruction of structural and functional proteins, resulting in skeletal muscle wasting [7]. These changes are highly pronounced in dialysis patients due to fatigue, inactivity, and the catabolic effect of the HD procedure itself [8–13].

There have been many approaches to treat sarcopenia, and exercise and nutritional support were effective as prevention and treatment strategies for sarcopenia [14], although study outcomes were inconsistent due to the differences in exercise dose, use of nutritional supplementation, and baseline nutritional status [15–17]. A review pointed that nutritional supplementation and exercise should be prescribed at the right patient (a patient who is at risk or with PEW and can tolerate and benefit from the exercise), right time (when the catabolism is at its peak or in between meals and when the patient’s stamina is the highest), and right dose (nutritional supplementation with amount and mixture that allow most efficacy and beneficial exercise intensity but not ineffective or even harmful), and recommended both interventions as a part of healthy lifestyle modification [18].

Protein servings are often deficient in the Korean diet. The Korea National Health and Nutrition Examination Survey (KNHANES) 2012–2016 data showed 40.5% and 59.9% of males and females older than 75 years took protein less than the estimated average requirements. Many patients receiving dialysis maintain the protein-restricted diet due to the fear of hyperkalemia and hyperphosphatemia. And several studies reported that protein intake was less than 1.0 g/kg/day in about half of HD patients [19, 20]. The typical dietary protein intake recommendation for ESRD patients is 1.2 g/kg/day [21]. However, some experts recommended a dietary protein intake of more than 1.8 g/kg/day in dialysis patients because they do not have to prevent CKD progression and are in a hypercatabolic state due to dialysis itself [22]. Protein supplementation during dialysis improved body composition and physical function and reduced inflammatory markers [23, 24]. Short-term oral energy or protein/amino acid supplementation improved nutritional status by increasing serum albumin levels and BMI without influencing serum potassium levels in dialysis patients in a meta-analysis [25]. In addition, renal-specific oral nutritional supplementation with a low-protein intake prevented a decline in nutritional status and quality of life without increasing the need for phosphorus binders [26].

Exercise is an anabolic-based strategy along with nutritional support, anabolic steroids, and growth hormones. Net protein catabolism occurs when protein and energy intake are inadequately low. However, resistance training during protein restriction counteracts impaired muscle cell metabolism [27]. Exercise improves muscle size, protein synthesis rate, neuromuscular function, insulin sensitivity, and inflammation associated with increased insulin-stimulated glucose and amino acid transport [3, 28].

However, physical inactivity is an important clinical problem among dialysis patients [27]. The Kidney Disease Outcome Quality Initiative (K/DOQI) and European guidelines recommend that nephrologists counsel and encourage dialysis patients to increase physical activity [29, 30]. Providing exercise therapy to patients who are unable to perform conventional dynamic training due to position limitations, hemodynamic instability during dialysis, low motivation, or fatigue is a major challenge [31]. HD patients, especially elderly patients, reported low levels of physical activity, which is associated with poor physical function and frailty [32].

Intradialytic exercise (IDE) can enhance or replace physical exercise in frail and exercise-hesitant HD patients. Neuromuscular electrical stimulation (NMES) induces muscle contraction by stimulating muscles or peripheral nerves, which prevents muscle atrophy due to central or peripheral muscle paralysis. NMES has the strength of application to exercise-hesitant patients. Recently, many studies have evaluated the feasibility and efficacy of intradialytic NMES. Intradialytic NMES of the quadriceps or gluteal muscles improved muscular strength, muscle cross-sectional area, functional capacity, and quality of life [33, 34]. Intradialytic NMES improved cardiorespiratory reserves (VO_2_ peak, VO_2_ anaerobic threshold) and muscle strength [31]. It also improved physical functioning and health-related QOL [35]. In addition, intradialytic NMES altered inflammatory markers, as it increased the levels of IGF-1, reduced the levels of IL-10, and reduced DNA damage in blood [36, 37]. Therefore, intradialytic NMES can also be a good option to enhance muscle strength and physical functioning as well as exercise compliance in HD patients, especially exercise-hesitant patients.

However, there has been no research about the effect of simultaneous nutritional support and NMES in dialysis patients. Therefore, we designed a 12-week trial comparing control, protein supplementation only, intradialytic NMES only, and intradialytic NMES combined with protein supplementation. In this study, we will evaluate the differential effectiveness of nutritional support and NMES during HD on muscle power, muscle mass, physical function, and QOL.

## Methods/design

### Study design

**Fig. 1:**
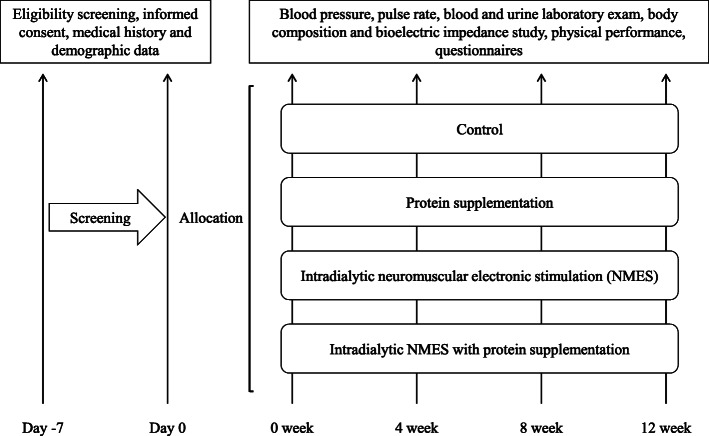
Clinical trial design

The study protocol was designed in accordance with the SPIRIT guidelines [38]. This is a 12-week, randomized controlled, parallel-group, multicenter trial of intradialytic NMES and protein supplementation for HD-dependent ESRD patients. Seventy-two patients receiving HD will be randomly assigned in a 1:1:1:1 ratio to the control, intradialytic NMES only, protein supplementation only, and intradialytic NMES combined with protein supplementation groups. Measurements will be scheduled for all patients at baseline and at follow-up every 4 weeks for 12 weeks; the measurements will include vital signs; blood and urine laboratory exams including total protein, albumin, total cholesterol, calcium, phosphorus, blood urea nitrogen (BUN), serum creatinine (SCr), total CO_2_, intact parathyroid hormone (PTH), highly sensitive C-reactive protein (hs-CRP), urine urea nitrogen/normalized protein catabolic rate (nPCR), and Kt/V; and body composition including bioelectric impedance study, physical performances, and questionnaires. Figure 1 presents a flowchart of the trial procedures.

The primary endpoint was the difference in hand grip and leg muscle strength at 12 weeks among the 4 treatment groups. Secondary endpoints included the following: muscle mass; physical performance, including the 10-m walking test and timed-up-and-go test; and questionnaires about QOL, physical activity, depression, and falls.

### Recruitment and population

A total of 72 patients with ESRD receiving HD will be recruited in four tertiary hospitals in Korea.

Subjects will be eligible for inclusion in this study only if all of the following criteria apply: (1) stable ESRD patients who have been receiving HD for 3 months or more, (2) patients with serum albumin less than 4.0 g/dl and are suspicious of having PEW, and (3) patients who can thoroughly understand the protocol.

Subjects will be excluded for any of the following reasons: (1) acute kidney injury; (2) uncontrolled hypertension, diabetes, or heart failure; (3) aspartate aminotransferase (AST) or alanine aminotransferase (ALT) level 3 times or higher than the upper normal limit of the participating hospital; (4) hematologic or solid organ malignancies under treatment; (5) active infection; (6) positive for HIV/AIDS; (7) history of ischemic heart disease, stroke, or deep vein thrombosis within 3 months before participation; (8) planning to receive kidney transplantation within 3 months after participation; (9) contraindication to electronic stimulation including implantable defibrillator; (10) skin lesions around the electrical stimulation spots; (11) allergy to protein supplements; (12) history of participation in another clinical study within 2 months prior to study enrollment and plans to participate in another clinical study; and (13) researcher’s decision regarding inappropriateness of enrollment in the study.

All patients who are eligible for this study will be asked by study investigators. They will receive oral and written information from the investigator. The investigator will obtain written informed consent and the patient will be randomized.

As the participation in the study is voluntary, all participants are free to withdraw their consent to participate in the study at any time and for any reason without any further treatment or any consequences. Moreover, participation of any participants could be terminated by an investigator at any time, if an investigator believes it is in the best interest of the participant. We shall document the reasons and circumstances of the study discontinuation in the clinical record form.

### Randomization and concealment

The experiment will be conducted by block randomization (block size of 4; stratified block randomization schedule for 4 institutions; each institution has a separate randomization schedule); the blind code will be computer-generated by a statistician of an external organization (Seoul National University Hospital Medical Research Collaborating Center) using SAS®, who had no contact with participants. Only an independent physician assistant at the central coordinating institution will have the pregenerated codes, and all other research members will be blinded. Researchers call a central coordinating office for each patient assignment. Participants will be randomly assigned to the control (random code A), intradialytic NMES (random code B), protein supplementation (random code C), or intradialytic NMES combined with protein supplementation (random code D) for 12 weeks according to their assigned code, after they agree to enroll in the study and sign the informed consent form.

### Interventions

#### Neuromuscular electrical stimulation

**Fig. 2:**
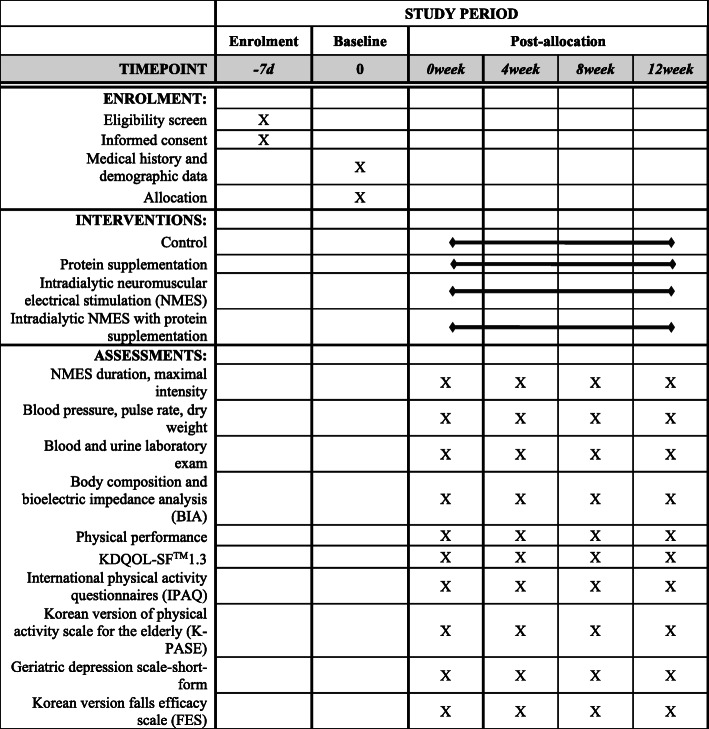
Summary of enrolment, interventions, and assessments at each study visit (SPIRIT)

NMES will be conducted three times a week during each dialysis session for 12 weeks. NMES will be performed as follows using a 4-channel functional electrical stimulation instrument (FES-1000/5000®, Stratec, Korea): impulse type: symmetrical biphasic, impulse width 400 μs, impulse frequency 60 Hz, and impulse on-off time 4–8 s. FES-1000 and FES-5000 are only different in batteries and provide the same electrical impulse. Participants will notify if there are any unpleasant symptoms. Impulse intensity will be individually adjusted to the maximum tolerated amperage for each stimulation session (impulse intensity range 0–100 mAp). Participants will be instructed to remain relaxed in a supine position with knees in extension during dialysis. Adhesive electrodes will be attached to a total of four areas of the bilateral lower extremities: vastus medialis: 3 cm above the upper border of the patella and 5 cm below the inguinal fold, vastus lateralis: 3 cm above the superior border of the patella and 5 cm below the inguinal crease toward the anterior superior iliac crest. NMES will be administered between 1 and 3 h after dialysis for 20 min during the first week and for an additional 2 min every week thereafter until 30 min (Fig. 2).

#### Protein supplementation

Protein supplementation will be provided and consumed at the beginning of every dialysis session or after the NMES. A total of 25 g of protein supplements including whey and soy proteins from Maeil Co. Ltd., Korea, i.e., renal-specific supplements (Mediwell®, protein 15 g, 400 kcal) and protein isolate powder (nutria-bridge protein powder®, protein 10 g), will be mixed together and taken.

### Outcomes

#### Primary outcomes

The primary outcomes are the hand grip and leg muscle strength of the HD patients in the different interventions, as assessed by using digital hand and leg dynamometers.

#### Secondary outcomes

Secondary outcomes include the difference between treatment arms and the change throughout the study period of (1) nPCR, (2) muscle mass through bioelectrical impedance analysis, (3) physical performance assessed by gait speed through a 10-m walking test and the timed-up-and-go test (3 m), and (4) questionnaire surveys. Questionnaire surveys included the Kidney Disease Quality of Life (KDQOL) short form, International Physical Activity Questionnaire (IPAQ), Korean version of Physical Activity Scale for the Elderly (PASE), Geriatric Depression Scale, and Korean version of Falls Efficacy Scale (FES).

### Measures

#### Biochemistry

We will collect biochemical data at baseline and follow-up every 4 weeks for 12 weeks, including hemoglobin, BUN/ SCr, calcium/phosphorus, intact PTH, total CO_2_, AST/ALT, total protein/ albumin, total cholesterol, hs-CRP, 24-h urine urea nitrogen/nPCR, and Kt/V.

#### Hand grip strength and leg muscle strength

Muscle strength will be assessed by using digital hand and leg dynamometers (T.K.K.5401 and 5710e/5715, Takei Scientific Instruments Co. Ltd., Niigata, Japan). Muscle strength will be collected at baseline and during the follow-up period at intervals of 4 weeks. Digital hand and leg dynamometers can measure from 5 to 100 kg. These dynamometers were used to evaluate muscle strength in several studies of elderly people [39–41].

A digital hand dynamometer (T.K.K. 5401) will be used to measure the hand grip strength (opposite the fistula side). Participants will measure the hand grip strength in two ways: sitting and standing. Participants will be seated, with the shoulder along the body and no rotation, 90° elbow flexion, and neutral flexion to measure sitting position hand grip strength. Participants will stand up with the shoulder along the body and with no rotation, elbow extension, and neutral flexion for standing position measurement. Participants will be encouraged to grasp strongly, three times at intervals of 1 to 2 min in each way.

A digital leg dynamometer (T.K.K. 5710e/5715) will be used to measure the leg muscle strength of the knee joint extension muscles (both sides) as directed in the manufacturer’s instructions. Participants will be seated, with 90° of knee flexion, and measurements will be repeated three times at intervals of 1 to 2 min.

### Body composition

We will collect anthropometric data, including weight, height, and circumferences of the waist, upper arm, and calf. Anthropometric data will be collected after the HD. Waist circumference will be measured at the midline level between the inferior margin of the ribs and the superior border of the iliac crest. Arm circumference will be measured with 90° elbow flexion at the midline level between the greater tubercle of the humerus and the olecranon of the ulna. Calf circumference will be measured at the thickest part with knees bent at 90° in the chair.

Muscle mass will be measured by using an InBody S10 (Seoul, South Korea), an instrument based on bioelectrical impedance analysis. InBody S10 can measure intracellular fluid, extracellular fluid, and water ratio and show muscle and lean body mass by body part. We will collect data including skeletal muscle mass (SMM), SMM/body weight (%), appendicular skeletal muscle mass (ASM), ASM/body weight (%), ASM/height^2^, lower extremity skeletal mass (LESM), LESM/body weight (%), LESM/height^2^, total fat mass, and percent body fat. This instrument was used in several studies evaluating body composition in HD patients [42, 43]. Participants will have their muscle mass evaluated within 30 min after HD.

Body composition will be collected at baseline and during the follow-up period at intervals of 4 weeks.

### Performance tests

#### 10-m walking speed test

A 10-m walking course should be open at least 60 cm from both ends and is comprised of a 6-m measurement course and a 2-m acceleration and deceleration zone. Participants walk with normal walking speed after the instruction of the examiner. Participants can use walking aids such as walking sticks or canes. We will adopt a dynamic start method. Participants start walking 2 m prior to the measurement point (acceleration zone) and ended 2 m after the measurement point (deceleration zone). Participants repeat the walking speed test 2 times, and faster data will be analyzed.

#### Timed-up-and-go test

Participants will be seated close to the back and stand and walk 3 m and turn around the marked point and return to the starting point after the instruction of the examiner. Participants repeat the timed-up-and-go test 2 times, and faster data will be analyzed.

### Questionnaire survey

#### Quality of life [44]

We will use the Kidney Disease Quality Of Life (KDQOL) short form version 1.3 to evaluate health-related QOL. The KDQOL short form was developed to evaluate the QOL of CKD and dialysis patients and is a self-report format. It has been widely used and validated. It consists of 43 kidney disease-related questions, 36 physical and mental health-related questions, and 1 general health state question. Scoring algorithms given in the user manual will calculate scores ranging from 0 to 100. The scores represent the percentage of the total possible score achieved, with 100 representing the highest QOL.

#### International Physical Activity Questionnaire (IPAQ) [45]

We will use the short self-administered version of the IPAQ that covers activity in the last 7 days. It comprises 7 items that are open-ended questions surrounding individuals’ last 7-day recall of physical activity. The IPAQ evaluates the usual physical activity that people perform as part of their everyday lives. It comprises job-related physical activity, transportation physical activity, housework, house maintenance, caring for family, recreating, exercise, and sport. Vigorous physical activities refer to activities that take hard physical effort and make breathing much harder than normal. Moderate activities refer to activities that take moderate physical effort and make breath somewhat harder than normal.

#### Korean version of the Physical Activity Scale for the Elderly (PASE) [46]

The Physical Activity Scale for the Elderly (PASE) is an easily administered and scored instrument that measures the level of physical activity in individuals aged 65 years and older. It comprises self-reported occupational, household, and leisure activity items over a 1-week period. The PASE scoring algorithm was derived from physical activity measured by movement counts from an electronic physical activity monitor, activity diaries, and self-assessed activity levels in a general population of non-institutionalized older persons. PASE scores are calculated from weights and frequency values for each of 12 types of activity. The sum of the PASE scores may range from 0 to 400 or more.

#### Depression (Geriatric Depression Scale) [47]

The Geriatric Depression Scale is a self-reported measure of depression in older adults. We will use the short form of the Geriatric Depression Scale, which is comprised of 15 items. This form can be completed in approximately 5 to 7 min. Of the 15 items, 10 indicate the presence of depression when answered positively while the other 5 are indicative of depression when answered negatively.

#### Korean version of Falls Efficacy Scale (FES) [48, 49]

The Falls Efficacy Scale assesses the perception of balance and stability during activities of daily living. It assesses fear of falling in the elderly population. A 10-item questionnaire estimate confidence in their ability to perform 10 daily tasks without falling as an indicator of how one’s fear of falling impacts physical performance. Each item is rated from 1 (very confident) to 10 (not confident at all), and per item ratings are added to generate a sum. Total scores can range from 10 (best possible) to 100 (worst possible). Lower scores indicate more confidence, and higher scores indicate lack of confidence and greater fear of falling.

### Informed consent

A trained research physician or investigator will introduce the trial to the patients and inform the patients of the details of the trial, including the use of NMES, protein supplementation, and adverse reactions. At the same time, informed consent in written form will be provided for patients to read. Only when patients fully understand the process of the trial, they can choose to participate voluntarily or give up. Patients who are disabled in reading or writing should be informed through their guardians or caregivers and sign informed consent on behalf of them.

### Harms

Adverse events are defined as any unfavorable and unintended signs, symptoms, or diseases, whether or not considered related to interventions. Every adverse event will be fully recorded on the case report form, and serious adverse events will be reported to the Institutional Review Boards concerned within 24 h after the recognition.

### Ancillary and post-trial care

Any study participant who wants to change or maintain the intervention after the completion of the study can receive the corresponding treatment as they want.

### Quality control, data management, and monitoring

This study is organized by a team comprised of a principal investigator and co-investigators who take part in the design and implementation of the study. For quality control purposes, all the researchers will be trained to be well-acquainted with the study protocol and to perform tests in one unified way before the recruitment of participants. The interventions will be performed by one expert at each hospital. All data will be put onto a computer with password protection by a staff blinded to the group allocation. The original forms of data will be archived securely at the central coordinating institution. All the investigators will be responsible for data monitoring, which is performed after each subject’s participation is completed. Monitoring includes assessment of the progress of the study and verification of accuracy as well as the completeness of recorded data. After completion of the study, we will submit the original data and results to IRBs of participating institutions, and after the publication of results, we will disclose original data and results to the public. Audits will be held annually by the IRBs of participating institutions.

### Sample size

Sample size estimation was based on detecting a mean between-group difference in leg muscle strength at the end of the study. Based on the results from an earlier study [34], the rationale mean (pooled standard deviation (SD)) for calculating the sample size was 24.82 (we chose the left leg SD, which was larger than the right leg SD of that study, to approach conservatively). Therefore, the means (SDs) of leg muscles in the NMES and control groups were 26.1 (24.82) and −8.3 (24.82), respectively.

Assuming a two-tailed hypothesis, an alpha value of 0.0083 due to the correction for type 1 error according to multiple tests, and a desired power of 80%, 13 participants are needed per group to complete the study. To allow a 25% dropout rate, we will initially recruit at least 72 participants.

### Statistical methods

The statistical processing will be performed with PASW advanced statistics (SPSS Inc, Chicago, IL) version 20.0 and according to an intention-to-treat principle. Data will be reported as the mean, standard deviation, and confidence intervals or in percentage frequencies. The Shapiro-Wilk test will evaluate the normal distribution of the variables. Evaluation variables, including muscle strength, performance tests, and questionnaire surveys, will be analyzed at baseline and after 4, 8, and 12 weeks by using paired *t*-tests or Wilcoxon signed-rank tests. Outcomes will be compared between groups by using two-sample *t*-tests or Wilcoxon rank sum tests according to normality. Analysis of covariance will also be conducted. The between-group differences in the outcome measures after the intervention will be calculated using repeated measure mixed models with patients as random effects, and group and time as fixed effects, and adjustments for baseline imbalances. The Spearman rank test or Pearson product-moment correlation coefficient analysis may be used to analyze the associations between clinical data and outcome measures. Statistical significance is set at a *p* value < 0.05. There is no plan for any interim analysis or stopping data collection. There are no planned subgroup analyses for any of the primary or secondary outcomes.

### Dissemination

The study findings will be presented at conferences and reported in peer-reviewed journals.

## Discussion

Sarcopenia occurs early and is more severe in CKD and ESRD patients. Reduced exercise capacity and functional decline due to sarcopenia are important because of their impact on QOL and morbidity [50]. Structural and functional declines in the cardiovascular system and skeletal muscles, which determine exercise capacity and VO_2max_, were less than 75% of the predicted values in CKD patients [31]. Approximately 71% of HD patients had a decrease in quadriceps force that was less than 2 SDs from the average of normal controls [6].

Nutritional support and exercise are the main strategies to prevent and treat sarcopenia. However, malnutrition is common in dialysis patients and many patients are exercise-hesitant.

Insufficient food contributes to PEW and an increased catabolic state, including persistent inflammation, metabolic acidosis, hormonal imbalance, and physical inactivity, leads to excessive muscle wasting [2, 51–53]. Malnutrition is considered a nontraditional cardiovascular risk factor in dialysis patients. Hypoalbuminemia is one of the most potent risk factors for mortality in HD patients [54]. Sarcopenia and low exercise capacity are predictors of morbidity and mortality and show reversed epidemiological tendencies in CKD and ESRD patients [2].

Proteolysis of muscles occurs when proteins or amino acids are required. Continuous and sufficient dietary protein intake is necessary because protein is not stored [11]. Leucine, branched-chain amino acid (BCAA), or β-hydroxy-β-methylbutyrate (HMB) is usually recommended for dietary protein intake in older people. Fast proteins, such as whey protein, may be beneficial compared to slow proteins, such as casein protein [28, 55]. In addition, animal-origin proteins can be better than plant-based proteins at accelerating protein synthesis, if we set aside the control of phosphorus and accompanying conditions in CKD and ESRD patients [28]. We expect protein supplementation including whey protein, as used in this trial, can improve muscle wasting.

Physical inactivity is an important clinical problem among CKD and dialysis patients [27]. Kidney Disease Improving Global Outcomes (KDIGO) recommends fully integrating exercise into the daily activities of CKD patients (≥ 30 min/day, ≥ 5 times/week) to improve cardiovascular fitness and tolerance [56]. The K/DOQI and European guidelines recommend that nephrologists counsel and encourage dialysis patients to increase physical activity [29, 30]. Physicians caring for CKD and ESRD patients should encourage patients to engage in physical activity such as aerobic, resistant, coordination, and flexibility exercises [32]. IDE can be a convenient option to ensure compliance and reduce uncomfortable dialysis-related symptoms such as restless legs and cramping [57]. IDE can enhance or replace physical exercise in frail and exercise-hesitant HD patients. Intradialytic cardiovascular and strengthening exercise improved oxygen uptake, inflammation, arterial compliance, muscle strength, physical activity, and psychosocial functioning. These types of exercise also improved the efficiency of dialysis (Kt/V) and solute clearance, including phosphorus, QOL, depression, and the hospitalization rate [58– 64]. IDE also increased blood pressure in patients with intradialytic hypotension, which has been associated with decreased cardiac output and increased mortality [65, 66]. Regular exercise also showed positive effects on nutritional markers such as serum albumin, prealbumin, and energy intake [27]. In addition, IDE combined with oral or parenteral nutrition enhanced amino acid uptake and protein content in the muscles of HD patients, indicating an improved anabolic effect of nutritional supplementation [67].

However, we often fail to encourage HD patients to perform intradialytic cardiovascular or resistance exercise because patients’ motivation to exercise is lacking. NMES can be a good alternative that can be offered to HD patients. It can be performed just according to the programmed schedule without patient volition. Several studies have proven the effectiveness of NMES in HD patients. However, there has been no research about the effect of simultaneous nutritional support and NMES in dialysis patients.

In summary, we expect that this study can provide guidelines for a new therapeutic option for HD patients who are unable or hesitant to exercise. Furthermore, this option could offer an opportunity to improve the physical function, QOL, and prognosis of HD patients.

## Trial status

The trial is ongoing and is currently recruiting participants. Recruitment was initiated on 26 October 2020 and is expected to be completed by the end of March 2022. The trial is based on a protocol version of 25 August 2019 (1.1). The trial is registered with https://cris.nih.go.kr (Clinical Research Information Service, CRIS), Korea, on 03 November 2020. The last update was posted on 03 November 2020.

## Data Availability

The final trial dataset will be only accessible for the principal investigator on reasonable request.

## Abbreviations

ASM: Appendicular skeletal muscle mass
BCAA: Branched-chain amino acid
CKD: Chronic kidney disease
ESRD: End-stage renal disease
FES: Falls Efficacy Scale
HD: Hemodialysis
HMB: β-Hydroxy-β-methylbutyrate
IDE: Intradialytic exercise
IPAQ: International Physical Activity Questionnaire
KDIGO: Kidney Disease| Improving Global Outcomes
K/DOQI: Kidney Disease Outcome Quality Initiative
KDQOL: Kidney Disease Quality Of Life
KNHANES: Korea National Health and Nutrition Examination Survey
LESM: Lower extremity skeletal mass
NMES: Neuromuscular electrical stimulation
nPCR: Normalized protein catabolic rate
PASE: Physical activity scale for the elderly
PEW: Protein-energy wasting
QOL: Quality of life
SD: Standard deviation
SMM: Skeletal muscle mass
